# Analysis of the Development and Thermal Properties of Chitosan Nanoparticle-Treated Palm Oil: An Experimental Investigation

**DOI:** 10.3390/nano15130972

**Published:** 2025-06-22

**Authors:** Varadharaja Kirthika, Chanaka Galpaya, Ashan Induranga, Amanda Sajiwanie, Vimukthi Vithanage, Kaveenga Rasika Koswattage

**Affiliations:** 1 Department of Food Science and Technology, Faculty of Applied Sciences, Sabaragamuwa University of Sri Lanka, Belihuloya 70140, Sri Lanka; rvkeerthikarv@gmail.com (V.K.); amanda@appsc.sab.ac.lk (A.S.); 2 Center for Nano Device Fabrication and Characterization (CNFC), Faculty of Technology, Sabaragamuwa University of Sri Lanka, Belihuloya 70140, Sri Lanka; chanakagalpaya@gmail.com (C.G.); ashaninduranga@tech.sab.ac.lk (A.I.); vimukkthi@tech.sab.ac.lk (V.V.); 3 Faculty of Graduate Studies, Sabaragamuwa University of Sri Lanka, Belihuloya 70140, Sri Lanka; 4 Department of Engineering Technology, Faculty of Technology, Sabaragamuwa University of Sri Lanka, Belihuloya 70140, Sri Lanka

**Keywords:** RBD palm olein, synthesized chitosan nanoparticles, nanofluids, energy saving, thermal properties

## Abstract

This study is an effort to optimize the thermal properties of refined, bleached, and deodorized (RBD) oil by incorporating bionanoparticles. This study investigates the impact on thermal conductivity and thermal diffusivity by incorporating chitosan nanoparticles (CS-NPs) at different temperatures with varying weight fractions of NPs. To the best of our knowledge, these synthesized CS-NPs from oyster mushrooms (*Pleurotus ostreatus*) and commercial marine-sourced CS-NPs are used for the first time to prepare nanofluids. These nanofluids offer high potential for industrial applications due to their biodegradability, biocompatibility, and nontoxicity. Fungal-sourced chitosan is a vegan-friendly alternative and does not contain allergic compounds, such as marine-sourced chitosan. The CS-NPs were synthesized using a chemical and mechanical treatment process at three different amplitudes, and CS-NPs at amplitude 80 were selected to prepare the nanofluid. Chitin, chitosan, and CS-NPs were characterized by the FTIR-ATR method, while the size and morphology of the CNs were analyzed by SEM. Thermal conductivity and thermal diffusivity of nanofluids and base fluid were measured using a multifunctional thermal conductivity meter (Flucon LAMBDA thermal conductivity meter) by ASTM D7896-19 within the temperature range 40–160 °C with step size 20. The thermal conductivity values were compared between commercial CS-NPs and synthesized CS-NPs treated with RBD palm olein with different weight percentages (0.01, 0.05, and 0.1 wt.%). It was confirmed that the thermal properties were enhanced in both kinds of nanoparticles added to RBD palm olein, and higher enhancement was observed in fungal-sourced CS-NPs treated with RBD palm olein. Maximum enhancement of thermal conductivity of commercial and synthesized CS-NPs treated with RBD palm olein were 4.28% and 7.33%, respectively, at 0.05 wt.%. Enhanced thermal conductivity of RBD palm olein by the addition of CS-NPs facilitates more effective heat transfer, resulting in quicker and more consistent cooking and other potential heat transfer applications.

## 1. Introduction

Palm oil can be considered a major frying oil due to its versatility; 80% of the oil is edible, while 20% is non-edible [1]. With a market share of over 36% worldwide, palm oil is the most-produced vegetable oil, increasing up to 74.7 million tons in 2020 [2]. Generally, most of the food industry uses refined red palm oil for frying to obtain a desirable color and flavor; it is used not only for frying purposes but also in food formulations, such as the production of butter, margarine, ghee, and confectionaries [1]. Producing palm oil is typically less expensive than producing the main alternative oils [3]. Therefore, palm oil is widely used all over the world in several industrial applications. Since it has wide applications in the food industry, the thermal properties of palm oil are an important factor in food processing, especially since thermal conductivity is important when frying, cooking, and baking. In the frying scenario, several chemical reactions can occur, such as oxidation, polymerization, and hydrolysis [4]. These chemical reactions lead to the degradation of oil and form degraded compounds that cause hazardous health effects on the human body. Therefore, using oil with better thermal stability is important when frying, cooking, and baking. Improving the thermal stability of oil is a new approach in the food industry, which carries several advantages.

A nanofluid is a fluid that contains nanoparticles that are less than 100 nm in particle size [5]. Nanofluids carry several advantages to industrial applications in electrical engineering, automotive, microelectronics, biomedical, and energy storage due to the exceptional properties of nanoparticles and their wide range of applications [6,7]. Nanofluids show significant heat transfer characteristics [8,9] due to the addition of nanoparticles to the base fluids. Generally, nanofluids are prepared by adding nanoparticles to any kind of base fluid, and those nanoparticles can be metal nanoparticles, such as Au, Ag, Cu, metal oxides, such as TiO_2_, Al_2_O_3_, CuO, Fe_2_O_3_, and carbon-based nanoparticles, such as graphene, fullerene, and carbon nanotubes [10]. Introducing nanofluids to the food industry is a novel approach that has several advantages. In the case of food applications, nanoparticles should be chosen to prepare nanofluids that are non-toxic, biocompatible, and biodegradable. Chitosan is a biopolymer that is derived from chitin. Chitin is a major component of the exoskeleton of crustaceans, insects, the cell wall of fungi, and fish scales [11]. Chitosan is approved as “Generally Recognized As Safe” (GRAS) by the United States Food and Drug Administration [12]. Due to the non-toxicity, biocompatibility, and biodegradability features of chitosan, it is applicable in food and pharmaceutical industries, biotechnology, agriculture and environmental engineering [13,14]. Generally, commercial chitosan is synthesized from the waste that is generated by seafood industries, such as shells of shrimps, crabs, lobsters, krill, and squid [15]. Since most chitosan is obtained from seafood waste, these marine-sourced chitosan can lead to allergic reactions due to the presence of tropomyosin, myosin light chains, and arginine kinase [16,17]. In contrast, fungal-based chitosan does not contain these allergic compounds, and it can be a vegan-friendly alternative [17].

Chitosan is known as a recyclable, inexhaustible, and renewable resource. Also, chitosan from different sources shows various physicochemical properties, and those properties lead to vast applications [18]. Due to the great functional and biological properties of fungal-sourced chitosan and its vegan appeal, there are a number of applications for it. The functional properties of fungal-sourced chitosan, such as high bacteriostatic, antimicrobial properties, film-forming ability, chelation, biodegradability, biocompatibility, and solubility, allow for vast applications of chitosan in the food industry [17,19]. For example, chitosan is used in wine production as a fining agent and preservative [20].

It is used as an additive, carrier, absorbent material, and for food as well as biomedical applications [21]. Improving the thermal properties of oil by the addition of chitosan nanoparticles is a novel and emerging application in the food industry.

In this study, marine-sourced chitosan nanoparticles (commercial) and fungal-sourced chitosan nanoparticles (synthesized) were selected as the nanoparticles, and refined, bleached, and deodorized (RBD) palm olein was used as the base fluid. This study compares the thermal conductivity, thermal diffusivity, and viscosity of chitosan-based nanofluids with RBD palm oil at different temperatures and different weight concentrations.

## 2. Materials and Methods

### 2.1. Materials

Chitosan nanoparticles (marine-sourced) were purchased from HIMEDIA (degree of deacetylation (DD%) ≥ 75%). RBD palm olein (Fortune brand) was purchased from the local market. Oyster mushrooms were purchased from the local market to synthesize chitosan nanoparticles. Hydrochloric acid (HCl) and sodium hydroxide (NaOH) were reagent grade and purchased from Sigma-Aldrich (St. Louis, MO, USA).

### 2.2. Experimental Methods

#### 2.2.1. Synthesis of Chitosan Nanoparticles

In the first step of the synthesis, 200 g of mushroom was dried at 60 °C. The dried mushroom sample was ground well. Dried ground mushroom powder was treated with 250 mL of 2M HCl solution to remove minerals, then the demineralization was carried out for 15 h at 60 °C. By the end of the demineralization, the sample was filtered through a filter paper by rinsing with distilled water. Then, the filtrate was removed, and the residuals were treated with 2M NaOH solution at 85 °C to remove the protein residues in the structure. After the 24 h NaOH treatment, the sample was washed with distilled water until reaching a neutral pH value. Finally, the sample was rinsed again with distilled water and kept in an oven at 50 °C until dried [18] to obtain chitin.

The synthesized chitin was refluxed with 60% NaOH at 130 °C for 4 h for chitosan output. After this process, the samples were filtered and washed with distilled water until reaching a neutral pH value. The obtained chitosan was kept in the oven at 50 °C for 24 h to dry [18].

**Figure 1:**
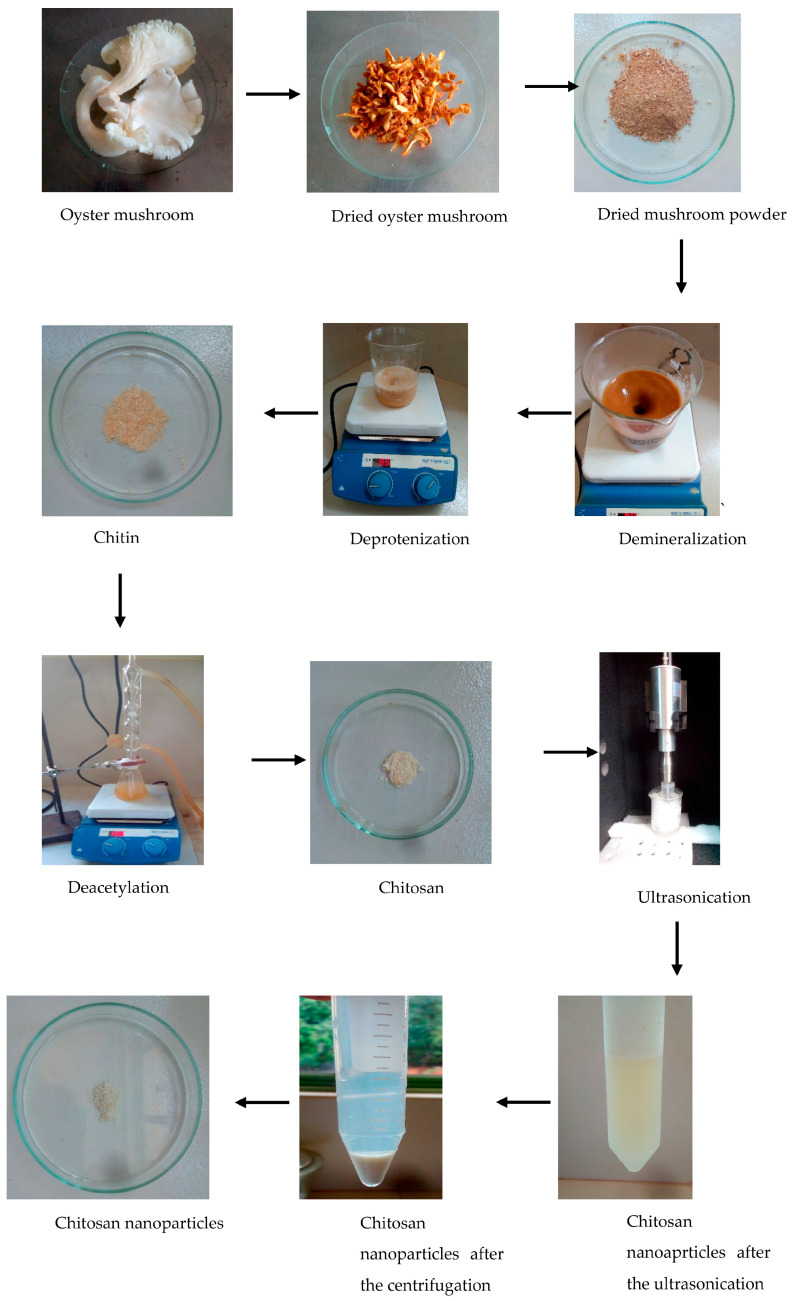
Synthesis of chitosan nanoparticles.

Then, the chitosan nanoparticles were prepared using direct ultrasonication of deacetylated chitosan dispersion. Before sonication, the concentration of the dispersion was adjusted to 0.1% (*w*/*v*) in a 100 mL glass beaker with distilled water. Ultrasonication was carried out using a probe-type ultrasonicator at 40, 60, and 80 amplitudes for 1 hr. After that, the centrifugation technique was utilized to separate the chitosan component (sediment) from water-soluble chitosan oligomers. Then, the sediment was removed and dried at 50 °C to obtain chitosan nanoparticles (Figure 1).

#### 2.2.2. Characterization of Chitin, Chitosan and Chitosan Nanoparticles

Fourier Transform Infrared–Attenuated Total Reflectance (FTIR-ATR) spectroscopy was analyzed using an FTIR spectrometer (Thermo Fisher Scientific, Madison, WI, USA) to confirm the chemical conformation of the synthesized chitin, chitosan and chitosan nanoparticles. The sample was placed on a diamond crystal and pressed using a minigrip device to ensure contact between the sample and diamond crystal. FTIR-ATR spectra were obtained in the wavelength range from 4000 to 400 cm^−1^ with 4 cm^−1^ resolution in the 32 scans at room temperature. The particle sizes of synthesized chitosan nanoparticles were analyzed using Zeiss EVO LS15 Scanning Electron Microscope (SEM) (Carl Zeiss SMT GmbH, Oberkochen, Germany) by placing the sample on carbon tape, and gold sputtering was performed for SEM imaging.

#### 2.2.3. Determination of Degree of Deacetylation

The FTIR method was used to determine the degree of deacetylation [19]. The degree of deacetylation in chitosan was measured using absorbance at 1655 cm^−1^ for amide-I and 3540 cm^−1^ for the OH group, and Equation (1) below was used to calculate it.

(1) $$Degree\;of\;deacetylation=\frac{A1655}{A3450}\times \frac{100}{1.33}$$

where “1.33” represents the ratio of A1655/A3450 for fully N-acetylated chitosan.

#### 2.2.4. Analysis of the Peroxide Value of Palm Oil

The oil sample was dissolved in glacial acetic acid–isooctane in a 3:2 ratio. Upon addition of excess potassium iodide, which reacts with the peroxides, iodine is produced. The solution was titrated with standardized sodium thiosulfate using a starch indicator. The peroxide value was calculated as shown in Equation (2).

(2) $$Peroxide\;value=\frac{\left(S-B\right)\times N}{W}\times 1000$$

where:

*Peroxide value* = mEq peroxide per kg of sample
*S* = volume of titrant (ml) for the sample
*B* = volume of titrant (ml) for blank
*N* = normality of Na_2_S_2_O_3_ solution (mEq/mL)

#### 2.2.5. Determination of Free Fatty Acid Percentage (FFA%)

An amount of 10 g of oil sample was measured, then 95% neutralized ethanol and phenolphthalein indicator were added. Then, the sample was titrated with NaOH, and the FFA% was calculated using Equation (3).

(3) $$\%\;FFA\;\left(as\;oleic\right)=\frac{V\times N\times 282}{W}\times 100\;$$

where:

*% FFA* = percent free fatty acid (g/100 g) expressed as Oleic acid
*V* = volume of NaOH titrant (mL)
*N* = normality of NaOH titrant (mol/1000 mL)
282 = molecular weight of oleic acid (g/mol)
*W* = sample mass (g)

#### 2.2.6. Preparation of Nanofluids

**Figure 2:**
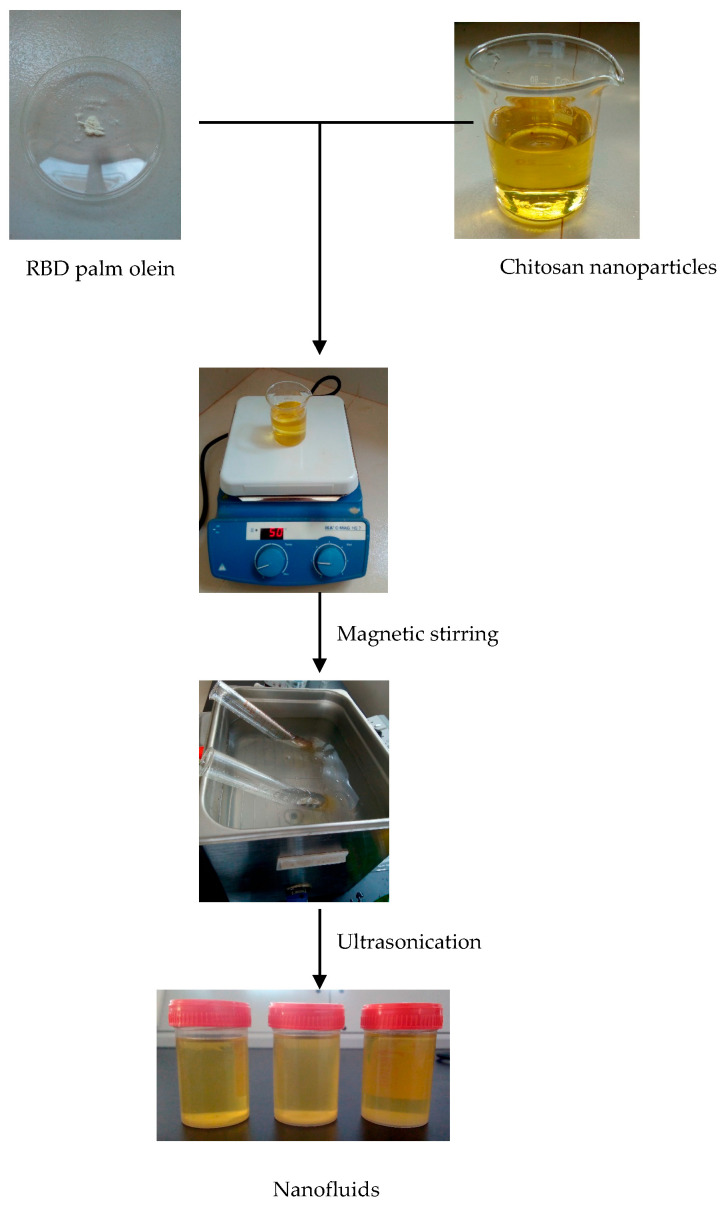
Preparation of nanofluids.

A two-step preparation method was used to utilize the base fluids with the nanoparticles (Figure 2). The synthesized chitosan nanoparticles and commercial nanoparticles were dispersed in the base fluid (RBD palm olein) with different weight fractions, such as 0.01 wt.%, 0.05 wt.%, and 0.1 wt.% which were calculated using Equation (4). The weight of nanoparticles was measured using a BSA224S-CW precision balance, Sartorius AG, Goettingen, Germany. After weighting, nanoparticles were dispersed within the oil at 60 °C and 500 rpm using a magnetic stirrer. The nanofluid samples were sonicated for 30 min in the bath-type ultrasonicator at 50 °C to eliminate particle agglomeration [10].

(4) $$Weight\;\%\;of\;nanofluid=\frac{m_{np}}{m_{np}+m_{bf}}\times 100$$

where:

*m_np_*—mass of nanoparticles
*m_bf_*—mass of base fluid

#### 2.2.7. Thermal Conductivity Measurement

The LAMBDA Thermal conductivity meter was used to measure thermal conductivity according to ASTM D7896-19 standardization [22]. It uses the hot wire transient method to measure the thermal properties, including thermal conductivity and thermal diffusivity. Equation (5) can be used to measure the thermal conductivity of the liquid.

(5) $$\lambda =\frac{q}{4\pi (\vartheta_{1}-\vartheta_{2})}ln(\frac{t_{1}}{t_{2}})$$

where λ is thermal conductivity (Wm^−1^ K^−1^), q is a constant heat stream (Wm^−1^), and ϑ is the temperature at a given time.

#### 2.2.8. Thermal Diffusivity Measurement

The thermal diffusivity of nanofluids was measured according to the ASTM D7896-19 standard method (Equation (6)).

(6) $$Thermal\;Diffusivity=\frac{k}{\rho C_{p}}$$

where K is the thermal conductivity, *ρ* is the density of the liquid, and Cp is the specific heat capacity at constant pressure.

#### 2.2.9. Viscosity Measurement

Both the kinematic and dynamic viscosity values of nanofluids were measured using the SVM 3001 Anton Paar viscometer (Austria). Kinematic viscosity was obtained using Equation (7).

(7) µ=kt

where k is the constant inherent to the viscometer used, 0.2407 mm^2^/s^2^, and t (s) is the liquid flow time through the capillary.

## 3. Results and Discussion

### 3.1. Quality Analysis of RBD Palm Olein

#### 3.1.1. Determination of Peroxide Value

Peroxide is a measure of the amount of peroxides formed in fats and oils through autoxidation and oxidation processes. Generally, it is a measure of the degree of initial oxidation of fats and oils.

According to The Food (Control of Quality) Act (1980), the maximum peroxide value of RBD palm olein is 2.0 meqO_2_/kg. According to the CODEX Alimentarius Commission, the peroxide value of oil used for consumption is 5.0 meqO_2_/kg. In our study, the initial peroxide value of RBD palm olein was 0.00 meqO_2_/kg (Table 1), which represents high-quality oil processing. Thorough refining, bleaching, and deodorizing procedures were used for this RBD palm olein in order to eliminate contaminants, free fatty acids, and other substances that may lead to oxidation. The results of the peroxide value of nanofluids were 0.00 meqO_2_/kg. Chitosan has an antioxidant property that inhibits autoxidation and peroxide formation. Therefore, chitosan nanoparticles did not react with RBD palm olein and did not contribute to peroxide formation in nanofluids.

#### 3.1.2. Determination of FFA Percentage

Statistically, the *p*-value is used to compare the significance level. If the *p*-value ≤ 0.05, there is a significant difference; if the *p*-value > 0.05, there is no significant difference. According to the results shown in Table 2, there was no significant difference between the FFA% of RBD palm olein and that of the nanofluids, since the *p*-value was 0.779, thus > 0.05. According to The Food (Control of Quality) Act (1980), the maximum FFA% of RBD palm olein is 0.1%. All the FFA% of nanofluids were within the range of the standard value. So, chitosan nanoparticles did not react with RBD palm olein, as they did not change the fatty acids profile of RBD palm olein. Therefore, the addition of nanoparticles to the RBD palm olein did not disrupt the quality of the oil.

### 3.2. Characterization of Chitin, Chitosan and Chitosan Nanoparticles

#### 3.2.1. FTIR Analysis of Chitin, Chitosan and Chitosan Nanoparticles

**Figure 3:**
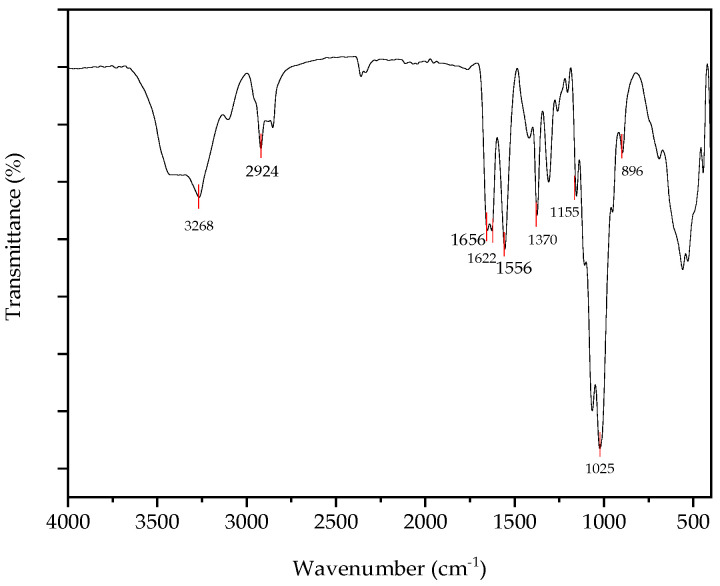
FTIR-ATR spectrum of chitin.

FTIR is a viable technique for rapidly comparing the properties of chitin, chitosan, and chitosan nanoparticles. FTIR analysis was conducted to confirm the synthesis of chitin and chitosan. Absorption bands in the regions of 896, 1155, and 1370 cm^−1^ were reported as chitin complexes extracted from certain mushroom varieties. In previous studies, absorption bands in the 890, 1155, and 1370 cm^−1^ regions were considered chitin complexes from some mushroom varieties [17]. Peaks at 896 were due to C-H bonding, and 1370 cm^−1^ was reported as C-H deformation of the glycosidic bond. The absence of absorbance in the region 1700–1740 cm^−1^ was reported as chitin extracted from *Pleurotus ostreatus*. The bands in the region of 1700–1740 cm^−1^ are represented as the presence of ester groups. So, their absence confirmed that chitin is free from fat [19]. Since the chitin was derived from *Pleurotus ostreatus*, the absence of absorbance at 3100 cm^−1^ was observed, which is a major difference between fungal chitin and marine chitin in the band at 3100 cm^−1^ [17]. Due to the influence of hydrogen bonding in the polymeric structure, the splitting of the peak into two characteristic bands at 1655 cm^−1^ and ~1620 cm^−1^ occurred [23]. Here, 1622 cm^−1^ was observed due to hydrogen bonds in the polymeric structure of chitin. The absorption peak at ~1553 cm^−1^ is due to the presence of N-H of the Amide II bond structure in the polymer [24]. The absorbance of 1556 cm^−1^ is observed in Figure 3 due to the presence of an amide II bond in the chitin.

**Figure 4:**
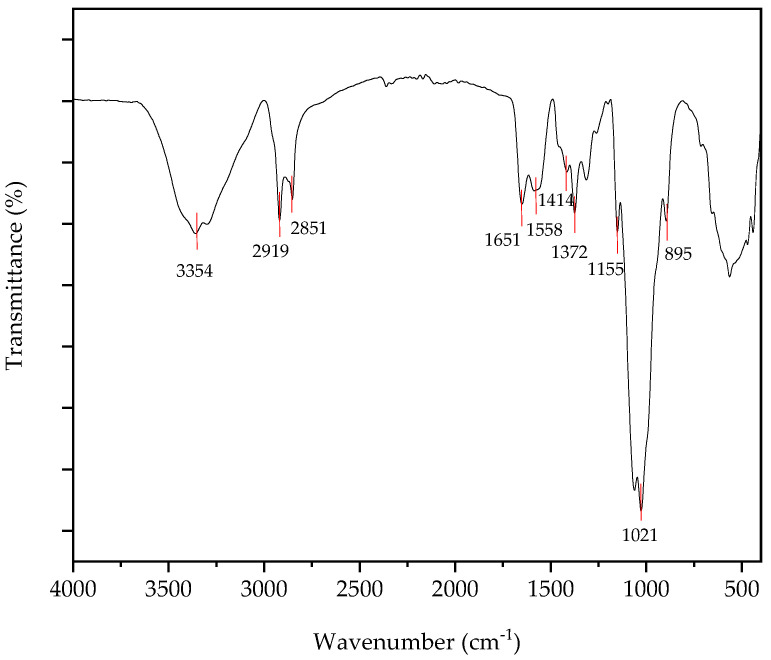
FTIR-ATR spectrum of chitosan.

FTIR spectrum of chitosan from mushrooms were analyzed, and the results indicate that these spectra are similar to other fungal sources as well as marine sources (Figure 4). A feature of mushroom-derived chitosan is a band in the 1600 cm^−1^ region associated with amine absorption, a band indicating OH wiggling at 3450 cm^−1^, and a CH stretching band in the 2875 cm^−1^ region [25]. For *Pleurotus ostreatus*, the peaks at 863.07 cm^−1^ can be attributed to the C-N stretching [18]. According to the results, chitosan derived from the *Pleurotus ostreatus* 896.38 cm^−1^ peak was observed, which represents the C-N stretching. Peaks at 1021.3 cm^−1^ for the spectrum may be assigned to the functional group of C-O stretching of the glucose molecule, while the C-H bonding of the side chain –CH_2_OH can be detected at 1401.5 cm^−1^ for the spectrum [17]. According to the results of the spectrum, 1026.72 cm^−1^ absorbance was observed, which represents the functional group of C-O stretching of the glucose molecule, while the C-H bonding of the side chain –CH_2_OH did not occur because absorbance at 1401.5 cm^−1^ of the spectrum was not detected. The peak at 1557.13 cm^−1^ in the relevant spectrum is indicative of an amide, but the peak at 3355.68 cm^−1^ for *Pleurotus ostreatus*-derived chitosan is indicative of free O-H groups. The peak at 1561.8 cm^−1^ is also an indication of an amide [19].

**Figure 5:**
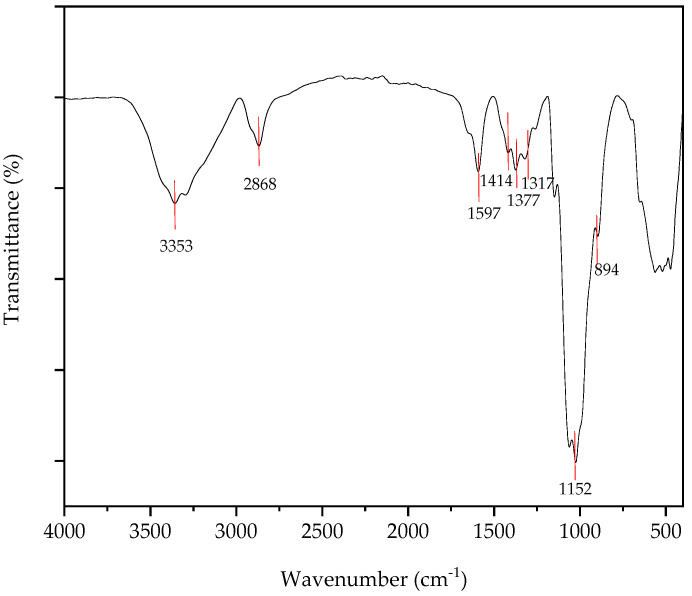
FTIR-ATR spectrum of chitosan nanoparticles.

The broad absorption band, which appears in the range of 3600–2800 cm^−1^, can be related to collective absorption by both N-H and O-H groups in the polymer. The broad absorption is a good indication of the strong hydrogen-bonded intermolecular structure. The absorption peak at ~1553 cm^−1^ in chitin disappeared, and a new peak emerged at 1597 cm^−1^ [26]. According to the spectrum (Figure 5), the peak at 1592.21 cm^−1^ represents amide II. The peak at 1597 cm^−1^ is due to the amide II functionality in the chitosan polymer, indicating the deacetylation of chitin.

#### 3.2.2. Degree of Deacetylation

The degree of deacetylation of chitosan nanoparticles was calculated as 85.21% using Equation (1).

#### 3.2.3. SEM Analysis of Chitosan Nanoparticles

**Figure 6:**
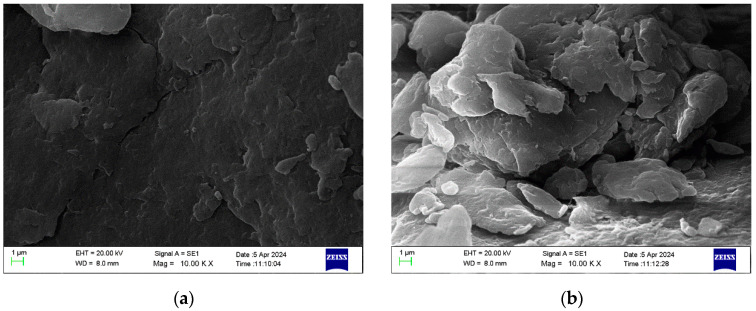

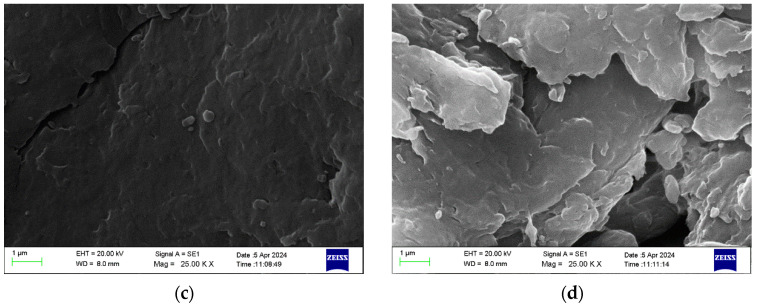
SEM images of chitosan nanoparticles synthesized at (**a**) 40 amplitudes, (**b**) 60 amplitudes, (**c**) 80 amplitudes, and (**d**) commercial chitosan nanoparticles.

Synthesized chitosan at 40, 60, and 80 amplitudes and commercial chitosan were analyzed by SEM to determine the particle sizes (Figure 6).

### 3.3. Determination of Thermal Conductivity of Chitosan Nanoparticle-Treated Nanofluids

**Figure 7:**
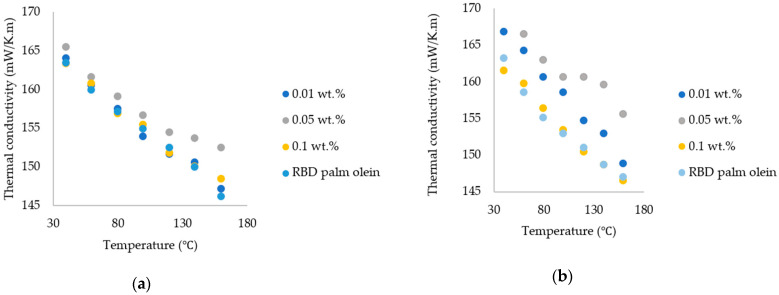
Thermal conductivity vs. temperature for (**a**) commercial (marine-sourced) chitosan-added nanofluids/RBD palm olein; and (**b**) synthesized (fungal-sourced) chitosan-added nanofluids/RBD palm olein.

Thermal conductivity changes of nanofluid as a function of temperature at different weight fractions are shown in Figure 7. The thermal conductivity of RBD palm olein and nanofluids was determined in the temperature range of 40 to 160 °C. The thermal conductivity of nanofluids was increased when the weight of nanoparticles increased in the RBD palm oil. According to the results, there was a significant difference between the thermal conductivity of the RBD palm olein and the nanofluids, since the *p*-value was 0.000 and thus < 0.05.

In a previous research study, higher enhancement in thermal conductivity was observed in carbon nanotubes [27]. Because of their strong van der Waals attraction and enormous surface energy, carbon nanotubes have a tendency to aggregate into groups. As a result, many strategies have been added to reduce the agglomeration of nanotubes, which are changing the surface chemistry of the tubes by means of metal coating, non-covalent adsorption by surfactant, and covalent (functionalization) through chemical modification. In previous studies, chitosan was used as a surfactant to disperse multi-walled carbon nanotubes (MWCNT) in the base fluid [28]. The thermal conductivity of nanofluids contained from 0.5 wt.% to 3 wt.% MWCNTs were enhanced from 2.3% to 13% [28]. This observation shows that chitosan contributes to the enhancement of thermal conductivity.

Chitosan nanoparticles are biopolymers that can enhance the dispersion and stability of the nanofluid, preventing agglomeration of the nanoparticles. The process of uniform dispersion guarantees that the nanoparticles are dispersed uniformly throughout the palm oil matrix, hence optimizing their contribution to the increase in heat conductivity. Thermal conductivity is improved as a result of the increased surface area, which facilitates more efficient contact with palm oil molecules. Van der Waals forces, hydrogen bonds, and electrostatic interactions are a few examples of these interactions that help with heat conduction [29].

The impact of chitosan nanoparticles on the thermal conductivity of RBD palm olein has a more significant effect on fungal-sourced chitosan nanoparticles than commercial marine-sourced chitosan nanoparticles. Since there is no previous research related to chitosan-added palm oil-based nanofluids, further research is needed on chitosan-based palm oils.

**Figure 8:**
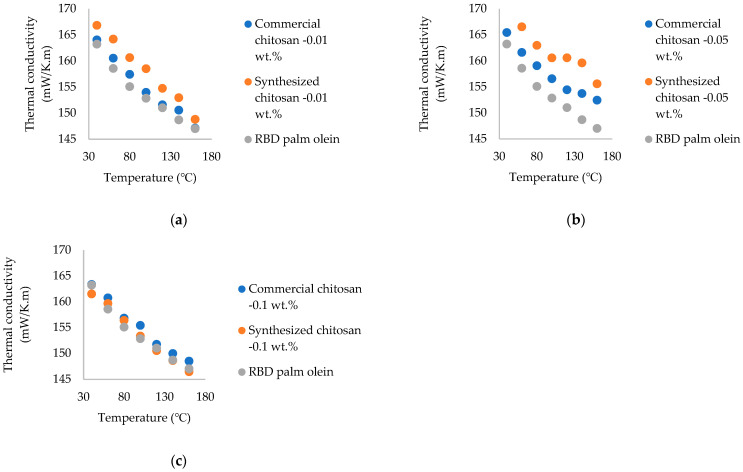
Thermal conductivity vs. temperature for different weight fractions of commercial (marine-sourced) and synthesized (fungal-sourced) chitosan nanoparticle-treated RBD palm olein; (**a**)—0.01 wt.%; (**b**)—0.05 wt.%; (**c**)—0.1 wt.%.

According to the results shown in Figure 8, there was a significant enhancement of thermal conductivity in the different nanofluid fractions. Maximum enhancement was observed at 140 °C. Therefore, these nanofluids are very suitable for frying purposes, as they can absorb heat very quickly at high temperatures.

While there was no study related to ours, when comparing the thermal conductivity enhancement of commercial chitosan and the synthesized chitosan nanoparticle-added RBD palm olein, the highest enhancement was observed in fungal-sourced chitosan nanoparticle-added palm olein. This was due to the purity of nanoparticles and the surface characteristics of nanoparticles. The DD% of commercial chitosan and synthesized chitosan nanoparticles was ≥75% and 85.21%, respectively. A higher DD% means more amino groups were available for interaction with palm olein molecules, potentially enhancing compatibility and dispersion. Mushroom-derived chitosan had higher DD% than marine-sourced which facilitates stronger interactions with palm olein, promotes electrostatic interactions with palm olein molecules and promotes thermal conductivity enhancement.

The higher surface area leads to the enhancement of the thermal conductivity of the oil. Synthesized nanoparticles had a larger surface area, which allowed for more contact points between chitosan nanoparticles and palm olein, promoting stronger interactions and better dispersion, resulting in enhanced thermal conductivity when dispersed in RBD palm olein.

According to the results, thermal conductivity enhancement was decreased at 0.1 wt.% throughout the temperature range of 40–160 °C. This observation was due to the higher amount of nanoparticles present in the oil. Therefore, precipitation was observed in the nanofluids, which represented the low stability of the nanofluid. So, no thermal conductivity enhancement was observed in 0.1 wt.%. If a surfactant or stabilizer is added to the nanofluids, it will improve the stability of the nanofluid and enhance the thermal conductivity, even with a higher amount of nanoparticles added to the oil. In previous studies, after the addition of surfactant to the nanofluids, a thermal conductivity enhancement was observed when carbon nanotubes were added to water [27]. A previous study also observed thermal conductivity enhancement by the addition of gum Arabic stabilizer to the carbon nanotube-added nanofluids [29]. Previous studies regarding thermal conductivity of nanofluids with different base fluids and different nanoparticle additives are represented in Table 3.

### 3.4. Comparison of Thermal Diffusivity of Commercial Chitosan Nanoparticle-Treated Nanofluids and Synthesized Chitosan Nanoparticle-Treated Nanofluids

**Figure 9:**
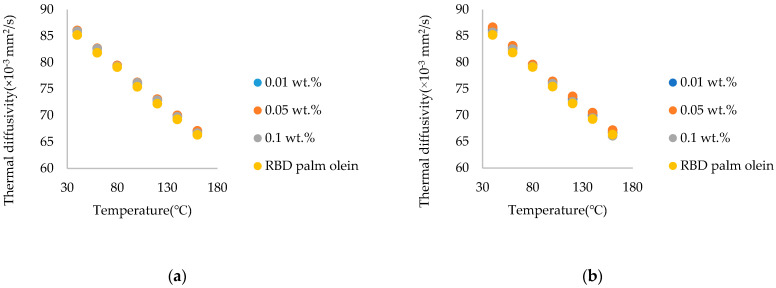
Thermal diffusivity vs. temperature for (**a**) RBD palm olein/commercial (marine-sourced) chitosan nanoparticle-treated RBD palm olein; and (**b**) RBD palm olein/ synthesized (fungal-sourced) chitosan nanoparticle-treated RBD palm olein.

According to the results shown in Figure 9, there was an enhancement in the thermal diffusivity of nanofluids compared to the RBD palm olein. Since the *p* value was <0.05 (*p*-value = 0.000), there were significant differences between the thermal diffusivity of RBD palm olein and the nanofluids with different weight fractions. Chitosan nanoparticles have a polymeric structure with amino and hydroxyl functional groups, which can interact with the molecules in RBD palm olein through hydrogen bonding and other interactions. These interactions might facilitate heat transfer and increase thermal diffusivity. Therefore, RBD palm olein’s thermal characteristics can be changed by adding chitosan nanoparticles, which may increase the thermal diffusivity of oil.

Similar to the commercial chitosan nanoparticle-treated RBD palm olein, thermal diffusivity was reduced at 0.1 wt.% in the oil of the fungal-sourced chitosan nanoparticle-treated RBD palm olein. This was due to the higher amount of nanoparticles present in the oil. So, to obtain better enhancement of thermal diffusivity even at a high wt.%, a surfactant should be added. The addition of surfactants improves the stability, which increases the thermal diffusivity of oil.

### 3.5. Comparison of the Viscosity of Commercial Chitosan Nanoparticles and Synthesized Chitosan Nanoparticle-Treated Nanofluids

**Figure 10:**
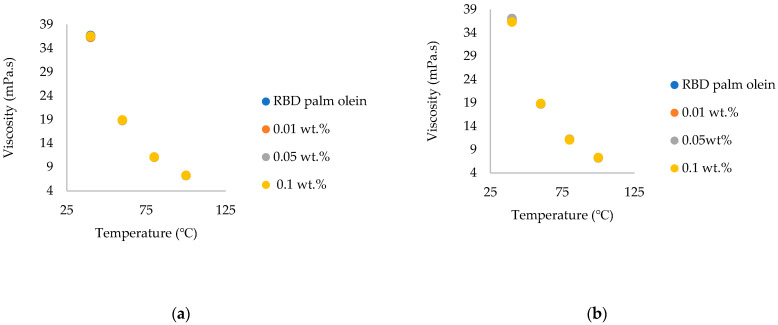
Viscosity of (**a**) commercial (marine-sourced) chitosan nanoparticle-treated nanofluids; and (**b**) synthesized (fungal-sourced) chitosan nanoparticle-treated nanofluids.

According to the results shown in Figure 10, the viscosity of nanofluids was increased when comparing the RBD palm olein to the addition of commercial and synthesized chitosan nanoparticles. Meanwhile, the viscosity values decreased when the temperature increased. These results were obtained because chitosan is a biopolymer that has a tendency to form hydrogen bonds with other chitosan molecules as well as with the molecules present in RBD palm olein. These intermolecular interactions can lead to the formation of a cohesive network structure within the oil, resulting in increased viscosity. In previous studies, kinematic viscosity for graphite-based nanofluids was tested at different temperatures and with varying weight percentages of nanoparticles. The results showed that the proportional viscosity of the nanofluids dropped as the temperature rose, and the viscosity of the nanofluids increased when the concentration of nanoparticles increased. The same results were observed in the previous study of palm oil-based nanofluids by the addition of TiO_2_, Al_2_O_3_ and AlN nanoparticles [42].

Since this was the first study analyzing the properties of chitosan-added nanofluids, a comparison between commercial chitosan and synthesized chitosan was conducted. When comparing synthesized chitosan with commercial chitosan, the viscosity of nanofluids was comparably higher in synthesized chitosan-added RBD palm olein than in commercial chitosan-added RBD palm olein. These results were due to fungal-sourced chitosan nanoparticle-added RBD palm olein-based nanofluids’ shear stress being higher than that of commercial chitosan (marine-sourced) nanoparticle-added RBD palm olein. This observation is explained by the Newtonian viscosity law. Due to higher viscosity, fluids offer greater resistance to flow, resulting in higher shear stress for a given shear rate. Conversely, low-viscosity fluids flow more easily and exhibit lower shear stress under the same conditions. Since a significant enhancement was observed in both types of nanofluids (Figure 10), it was not considered a higher enhancement. So, the addition of chitosan nanoparticles to the RBD palm olein did not have much impact on its viscosity.

## 4. Conclusions

In this study, the thermal properties of commercial chitosan (marine-sourced) and synthesized chitosan (fungal-sourced) nanoparticle-treated RBD palm olein were determined. These nanofluids ensure the minimization of energy consumed for cooking and minimize the formation of hazardous substances in the oil, especially during frying.

(a) The highest thermal conductivity enhancement was observed at 140 °C at 0.05 wt.% of synthesized and commercial chitosan nanoparticles, while at 160 °C, it was at 7.33% and 4.28%, respectively. Better thermal conductivity enhancement was observed throughout the temperature range of 40–160 °C. Fungal-sourced chitosan nanoparticles showed higher enhancement than marine-sourced chitosan nanoparticles. Lower enhancement was observed in 0.1 wt.% due to the higher amount of nanoparticles present in the oil. Therefore, the optimum wt.% to add chitosan nanoparticles to oil to obtain a higher enhancement of thermal conductivity is 0.05 wt.%.
(b) The thermal diffusivity of both commercial chitosan and synthesized chitosan nanoparticle-treated RBD palm olein was enhanced. Comparing marine–sourced with fungal-sourced, fungal-sourced chitosan increased thermal diffusivity more than marine-sourced chitosan.
(c) Observing the viscosity of commercial chitosan and synthesized chitosan nanoparticle-treated RBD palm olein, the viscosity of nanofluids increased due to the increase in the weight of nanoparticles in both cases. Viscosity enhancement by the synthesized chitosan nanoparticles was higher than by the commercial ones, and these enhancements were in an acceptable range for food applications. The findings of this novel study provide a dataset for future research on edible nanofluids, which can be applicable in the food industry. Future research work should focus on determining the biochemical changes, stability and rheological modeling of the nanofluids.

## Figures and Tables

**Table 1:** Peroxide value of RBD palm olein and nanofluids.

Sample	Weight Fraction of Nanoparticles (%)	Peroxide Value (meqO_2_/kg)
RBD palm olein (Initial)	-	0.00
Commercial chitosan (marine-sourced) Nanoparticle-added RBD palm olein	0.01	0.00
	0.05	0.00
	0.1	0.00
Synthesized chitosan (fungal-sourced) nanoparticle-added RBD palm olein	0.01	0.00
	0.05	0.00
	0.1	0.00

**Table 2:** FFA% of RBD olein and the nanofluids.

Sample	Weight Fractions (%)	FFA%
RBD palm olein (Initial)	-	0.10467 ± 0.0124 ^a^
Commercial chitosan (marine-sourced) nanoparticle-added RBD palm olein	0.01	0.10490 ± 0.0121 ^a^
	0.05	0.10430 ± 0.0128 ^a^
	0.1	0.097667 ± 0.0006 ^a^
Synthesized chitosan (fungal-sourced) nanoparticle-added RBD palm olein	0.01	0.097300 ± 0.0006 ^a^
	0.05	0.10483 ± 0.0122 ^a^
	0.1	0.097033 ± 0.0001 ^a^

Additionally, ‘^a^’, ‘^b^’ and ‘^c^’ represents the significant different between each value. Since all the values have same letter ‘^a^’, which statistically proved that there is no significant different between each FFA%.

**Table 3:** Thermal conductivity results of previous oil-based nanofluids.

Base Fluid	Nanoparticle	Concentration	Maximum Enhancement	References
Vegetable oil	Hexagonal boron nitride	0.02–0.1 vol.%	14%	[6]
Transformer oil	TiO_2_	0.002–0.012 vol.%	4.2%	[10]
Coconut oil	TiO_2_	0.002–0.012 vol.%	1.4%	[10]
Kerosene	Al_2_O_3_	0.05–0.5%	1.22%	[30]
Engine oil	MWCNT	0.1–0.5 wt%	1.227%	[31]
Mineral oil	Diamond	0–1.9 vol %	11%	[32]
Gear oil	Cu	0.11 and 2.0%	24%	[33]
Transformer oil	AlN	0.5 vol.%	8%	[34]
Transformer oil	Al_2_O_3_	4 vol.%	20%	[34]
Pongamia oil methyl ester (POME)	Exfoliated hexagonal boron nitride (Eh-BN)	0.01 wt.%	22.65%	[35]
Mineral oil	Eh-BN	0.01 wt.%	3.9%	[35]
Soybean oil (SO), coconut oil (CO), and palm oil (PO)	Al_2_O_3_ and TiO_2_	0.2 wt%, 0.4 wt% and 0.6 wt%	125.3% for PO 23.3% for SO 14.1% for CO	[36]
Transformer oil	Amorphous graphene (a-GS)	0.0012 wt%, 0.0025 wt%, 0.005 wt% and 0.01 wt%	30%	[37]
Purified aged transformer oil	SiO_2_, Al_2_O_3_, and TiO_2_	0.1 vol%	20.83%	[38]
Palm olein oil	ZnO	0.0025 g/L, 0.04 g/L and 0.14 g/L	59.5%	[39]
Palm fatty acid ester (PFAE)	ZnO	0.0025 g/L, 0.04 g/L and 0.14 g/L	27%	[39]
Heat transfer oil (LD320, heavy alkylbenzene	Graphite	1.36 vol.%	36%	[40]
Engine oil	TiO_2_	0.01 wt.%	4.5%	[41]
Engine oil	Fe_2_O_3_	0.01 wt.%	3.9%	[41]
RBD palm olein	Synthesized chitosan (Fungal-source)	0.01–0.1 wt.%	7.33%	Present study
RBD palm olein	Commercial chitosan (Marine-source)	0.01–0.1 wt.%	4.28%	Present study

## Data Availability

Data are contained within the article.

## Abbreviations

The following abbreviations are used in this manuscript:

RBD	Refined, bleached, deodorized
GRAS	Generally recognized as safe
CS-NP	Chitosan nanoparticle
DD	Degree of deacetylation
MWCNT	Multi-walled carbon nanotubes
Eh-BN	Exfoliated hexagonal boron nitride
SO	Soybean oil
CO	Coconut oil
PO	Palm oil
